# Glucose-Sensing Transcription Factor MondoA/ChREBP as Targets for Type 2 Diabetes: Opportunities and Challenges

**DOI:** 10.3390/ijms20205132

**Published:** 2019-10-16

**Authors:** Ziyi Song, Hao Yang, Lei Zhou, Fajun Yang

**Affiliations:** 1 College of Animal Science and Technology, Guangxi University, Nanning 530004, China; 2 Departments of Medicine and Developmental and Molecular Biology, Albert Einstein College of Medicine, Bronx, NY 10461, USA; 3 Division of Medical Genetics, Department of Pediatrics, Université de Montréal and CHU Sainte-Justine, Montreal, QC H3T 1C5, Canada

**Keywords:** glucose, MondoA, ChREBP, insulin resistance, type 2 diabetes, metabolism, skeletal muscle, liver, adipose tissue, pancreas

## Abstract

The worldwide increase in type 2 diabetes (T2D) is becoming a major health concern, thus searching for novel preventive and therapeutic strategies has become urgent. In last decade, the paralogous transcription factors MondoA and carbohydrate response element-binding protein (ChREBP) have been revealed to be central mediators of glucose sensing in multiple metabolic organs. Under normal nutrient conditions, MondoA/ChREBP plays vital roles in maintaining glucose homeostasis. However, under chronic nutrient overload, the dysregulation of MondoA/ChREBP contributes to metabolic disorders, such as insulin resistance (IR) and T2D. In this review, we aim to provide an overview of recent advances in the understanding of MondoA/ChREBP and its roles in T2D development. Specifically, we will briefly summarize the functional similarities and differences between MondoA and ChREBP. Then, we will update the roles of MondoA/ChREBP in four T2D-associated metabolic organs (i.e., the skeletal muscle, liver, adipose tissue, and pancreas) in physiological and pathological conditions. Finally, we will discuss the opportunities and challenges of MondoA/ChREBP as drug targets for anti-diabetes. By doing so, we highlight the potential use of therapies targeting MondoA/ChREBP to counteract T2D and its complications.

## 1. Introduction

Type 2 diabetes (T2D) is a chronic metabolic disease which affects more than 370 million people worldwide [1]. It is associated with high incidence of developing several medical complications, including but not limited to cardiovascular disease, kidney disease, some types of cancer, and vision problems [2,3]. T2D is one of the leading causes of global morbidity and mortality due to this [4]. Despite great progress that has been made in the understanding of the pathophysiologic mechanisms of T2D in recent years, the incidence and prevalence of the disease continues to rise globally, and the affected population is estimated to be as high as 570 million by 2040 [1]. T2D thereby places considerable socioeconomic pressures on the individual and huge costs to global health economies in the present and future [5]. The reasons for the escalating epidemic of T2D include being overweight or obese, sedentary lifestyle, and increased consumption of unhealthy diets, as well as refined grains and sugar-sweetened beverages, which are all related to economic globalization and urbanization [2,3].

At the cellular and molecular levels, T2D is characterized by relative insulin deficiency caused by pancreatic β-cell dysfunction and insulin resistance (IR) in target organs [1]. Although the specific mechanisms of T2D pathogenesis are still missing to date, the proposed models are as follows [6,7]. Normally, pancreatic β-cells produce insulin to maintain postprandial blood glucose level through increasing glucose disposal by insulin-sensitive organs (e.g., the skeletal muscle, liver, and adipose tissue), and meanwhile inhibiting hepatic glucose production. However, under conditions of chronic energy overload, ectopic lipid accumulation causes IR in the skeletal muscle and liver, leading to the impairment of glucose uptake and glycogen synthesis but promotion of hepatic de novo lipogenesis (DNL) and hyperlipidemia. In parallel, the overexpansion of white adipose tissue (WAT), especially the visceral adipose tissue (VAT), induces the production of pro-inflammatory adipokines (e.g., monocyte chemoattractant protein-1 (MCP-1) and tumor necrosis factor-α (TNFα)), which subsequently recruit the macrophage infiltration into the WAT, leading to the WAT IR and elevation of lipolysis. Then, the WAT disorder further increases hepatic triglyceride synthesis, hyperlipidemia, and hepatic gluconeogenesis, promoting fasting and postprandial hyperglycemia. In response, pancreatic β-cells initially compensate for IR by hypersecretion of insulin. However, as the disease progresses over time, the β-cell compensation fails due to β-cell apoptosis which is caused by chronic hyperglycemia and its complications, and eventually, T2D ensues.

Even though the modification of lifestyle, including weight loss, increasing physical activity and adopting a healthy diet [8,9], have been proved to be a practical strategy for the management of T2D, the anti-T2D drugs are still strongly needed. Not only patients who are unwilling to change their lifestyle require pharmacological treatment, but the progressive nature of T2D (relentless loss of functional β-cell mass) also constitutes a major need for novel antidiabetic agents that hold potentials of arresting this process. Currently, the available anti-T2D drugs (e.g., metformin) or the strategies of developing new drugs usually aim to increase pancreatic β-cell insulin production or systemic insulin sensitivity. However, so far, only small numbers of gene candidates, e.g., peroxisome proliferator-activated receptor-γ (PPARγ), have been identified and applied in clinical practices [10]. Notably, studies in recent years have shown that the glucose-sensing transcription factor MondoA/carbohydrate response element-binding protein (ChREBP) is emerging as a potential target to treat T2D [11,12,13,14]. To advance our knowledge of MondoA/ChREBP, this review will summarize the recent findings concerning the respective regulation and functions of MondoA and ChREBP in each metabolic organ (i.e., the skeletal muscle, liver, adipose tissue, and pancreas), with an emphasis on its challenges and opportunities in the treatment of T2D.

## 2. Glucose Sensors: MondoA vs. ChREBP

Glucose is used almost universally as the preferred carbon and energy source [15]. Breakdown of this major cellular nutrient provides both a source of the starting material for the synthesis of all major classes of biomolecules, and also ATP production through glycolysis and oxidative phosphorylation. Given the importance of glucose in metabolism, cells have evolved mechanisms to sense and utilize this crucial fuel in their environment. In mammalian cells, glucose or simple carbohydrates are sensed by several mechanisms, of which the MondoA/ChREBP transcription factor has been revealed to be at the core in linking glucose metabolism to downstream gene transcription [16,17,18]. Hence, derangements in this glucose-sensing pathway in mammalian cells often contribute to pathological conditions such as obesity, fatty liver, and T2D in both animals and humans [17,19,20,21].

### 2.1. Overview of the Intrinsic Regulation of MondoA/ChREBP Activity

MondoA (also termed as Mlx (Max-like protein x) -interacting protein, MLXIP) and its paralog ChREBP (also known as MondoB or Mlx-interacting protein-like (MLXIPL)) were discovered a decade ago when researchers tried to figure out the molecular mechanisms of intracellular glucose sensing [22,23]. Almost at the same time, Mlx, a binding partner of Mad and Mnt proteins [24,25], was found to be the common heterodimerization partner of both MondoA and ChREBP [23,26]. Later, the MondoA/ChREBP-Mlx complexes were identified as a conserved regulator of glucose metabolism from yeast to mammals [18,27,28,29].

**Figure 1:**
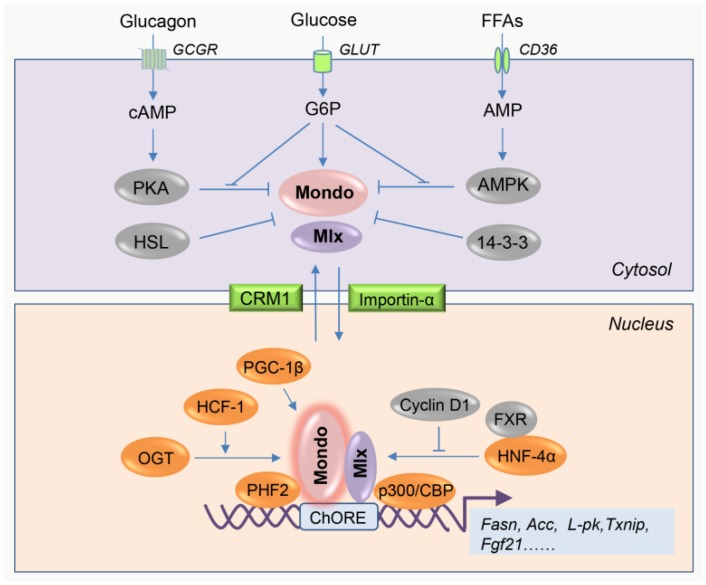
Regulation of MondoA/carbohydrate response element-binding protein (ChREBP) (Mondo) activity by nutrients and associated regulatory proteins. The activity of MondoA/ChREBP is dependent on its subcellular localization. Inactive state (upper panel): Under fasting conditions, intracellular cAMP and AMP activates protein kinase A (PKA) and AMP-activated protein kinase (AMPK) upon elevated glucagon release and fatty acids uptake, respectively. Then, the activated PKA and AMPK phosphorylate MondoA/ChREBP at different residues, consequently inhibiting the heterodimerization with Mlx and subsequent nuclear translocation. Besides, cytosolic protein 14-3-3 interacts with Mondo proteins and also blocks Mondo nuclear import. Particularly, in adipocytes, hormone-sensitive lipase (HSL) physically binds ChREBP-α and retains ChREBP-α in the cytosol, also limiting ChREBP activity. When under high glucose conditions, the intracellular glucose is phosphorylated into glucose-6-phosphate (G6P), a substrate which can be further used for producing other metabolites, such as xylulose 5-phosphate (Xu5P) and fructose-2,6-bisphosphate (Fru-2,6-P2). Thereafter, G6P and its certain derivatives induce a conformational change of Mondo, followed by the formation of Mondo-Mlx complexes, which then translocate to the nucleus through nuclear shuttling factor importin-α. Active state (lower panel): In the nucleus of hepatocytes, hepatocyte nuclear factor (HNF)-4α and peroxisome proliferator-activated receptor-γ coactivator (PGC)-1β physically interact with ChREBP and enhance its transcriptional activity. In contrast, cyclin D1 and farnesoid X receptor (FXR) inhibit ChREBP activity through suppression of HNF-4α function and dissociation of ChREBP from the p300/CREB-binding protein (CBP) transcriptional complex, respectively. Besides, host cell factor (HCF)-1 binds ChREBP and then recruits *O*-linked *N*-acetylglucosamine transferase (OGT) to ChREBP, stimulating ChREBP *O*-GlcNAcylation and activation. In parallel, HCF-1 also recruits PHD finger protein (PHF)2 for epigenetic activation of lipogenic gene promoters, further promoting ChREBP-dependent gene transcription. The arrow means the activation or promotion of the function, and the T-bar means the inhibition of the function.

These complexes play actions principally in two steps, including glucose metabolites-induced nuclear translocation and activation of target gene transcription by coordination with cofactors (Figure 1). A detailed representation of the activations has been presented elsewhere [16,17]. Briefly, under fasting conditions, all the components of the complexes are inactive due to their cytoplasmic localization. However, in response to carbohydrate feeding, the increased levels of glucose intermediate metabolites, e.g., glucose-6-phosphate (G6P) [29,30,31] and fructose-2,6-bisphosphate (Fru-2,6-P2) [32,33], might bind a specific recognition motif of MondoA/ChREBP, which may cause an allosteric conformational change of MondoA/ChREBP, that leads to MondoA/ChREBP recruitment of transcriptional partners (i.e., Mlx). Subsequently, MondoA/ChREBP-Mlx complexes translocate into the nucleus, where they bind the conserved carbohydrate response element (ChoRE) motif presented on promoters of the glycolytic (e.g., *L-pk* [22]), lipogenic (e.g., *Acc*, *Fasn*, *Scd1,* and *Elovl6* [28]) and other (e.g., *Fgf21* and *Txnip* [34,35]) genes, and then activate their transcription through coordination with other coregulators.

So far, several types of regulators have been identified to be involved in the activation or inhibition of MondoA/ChREBP-Mlx complexe formation or activities (Figure 1). They include post-translational modification enzymes (e.g., protein kinase A (PKA) [36,37], AMP-activated protein kinase (AMPK) [38,39], and *O*-linked *N*-acetylglucosamine (*O*-GlcNAc) transferase (OGT) [40]), nuclear shuttling factors (e.g., 14-3-3 protein [41,42], chromosomal region maintenance 1 (CRM1) [43], and importins [44]), nuclear receptors (e.g., hepatocyte nuclear factor (HNF)-4α [45] and farnesoid X receptor (FXR) [46]), transcription cofactors (e.g., peroxisome proliferator-activated receptor-γ coactivator (PGC)-1β [47]), epigenetic enzymes (e.g., p300/CREB-binding protein (CBP) [48] and PHD finger protein 2 (PHF2) [49]), accessory proteins (e.g., host cell factor (HCF)-1 [50] and cyclin D1 [51]), and even lipases (e.g., hormone sensitive lipase (HSL) [52]). These proteins are implicated in the regulation of all aspects of MondoA/ChREBP-Mlx complexes, including nucleo-cytosolic trafficking, DNA binding ability, transactivation activity, message RNA transcription, and protein stability. Their detailed functions are summarized by other authors [17,53].

### 2.2. Differences between MondoA and ChREBP

Despite the overall structural and functional similarities between MondoA and ChREBP (reviewed in other works [16,17]), there are differences (Table 1). First, despite both transcription factors have widespread expression, under normal physiological conditions, MondoA expression is most abundant in skeletal muscle where it mainly regulates the glycolytic pathway [23,54], while ChREBP expression predominates in the liver and adipose tissue (with moderate expression in the kidney, skeletal muscle, and small intestine) and primarily regulates lipogenesis [55,56], indicating MondoA-Mlx and ChREBP-Mlx may represent glucose sensors in different organs. Of note, the differences in downstream target genes are not simply due to differences in their expression patterns, but also due to the refined structural differences as each interacts with specific promoters [57]. Second, under basal conditions, MondoA locates to the outer membrane of the mitochondria [54], while ChREBP remains in the cytosol [37], indicating they play different roles under glucose-deprived conditions. Although no evidence is reported to date, the mitochondria-localized MondoA is probably involved in the regulation of mitochondria morphogenesis or respiration activity, which could be explored in future studies. Third, it has been reported that PKA signaling inhibits the activities of both MondoA and ChREBP through phosphorylation, but interestingly the phosphorylation sites are not conserved between them [58,59], suggesting their post-translational modifications are different. Last but not least, ChREBP has two isoforms, i.e., ChREBP-α (the full-length ChREBP) and recently identified ChREBP-β (a constitutively activated isoform lacking the low glucose inhibitory domain) [60]. In contrast, only the full-length isoform of MondoA is reported to date. Whether MondoA also has a constitutive activated isoform is still waiting to be confirmed. It is noteworthy that, during the last decade, ChREBP has gained more attention than MondoA in the fields, thus it is possible that as more understanding of MondoA develops in the future, the more differences between MondoA and ChREBP will be revealed.

## 3. Muscle MondoA: A Negative Regulator of Insulin Sensitivity

**Figure 2:**
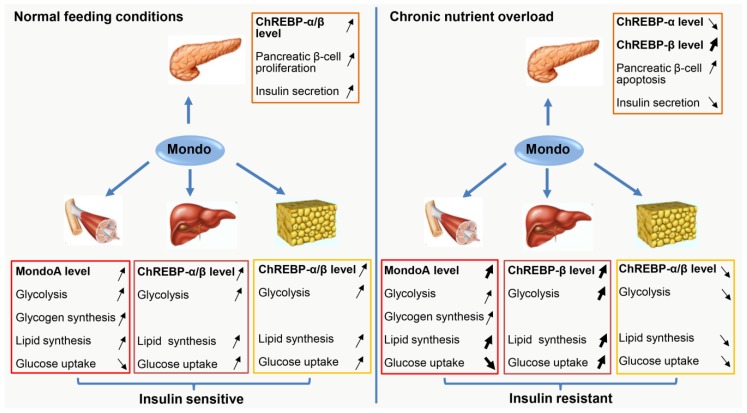
The roles of MondoA/ChREBP in metabolic tissues under normal feeding conditions and chronic nutrient overload. In response to normal carbohydrate diet (left panel), MondoA/ChREBP expression is induced in all metabolic tissues (including the pancreas, skeletal muscle, liver, and adipose tissue), which contributes to adaptive pancreatic β-cell proliferation and increased insulin production, and meanwhile, which also promotes glucose disposal by the muscle, liver, and adipose tissue through enhancing glycolysis, lipid synthesis, as well as glycogen synthesis. All these metabolic alterations guarantee whole-body insulin sensitivity and glucose homeostasis. However, when the body is exposed to chronic nutrient overload (right panel), the response of MondoA/ChREBP expression is not unified among the metabolic tissues. Specifically, in pancreatic β-cells, ChREBP-β expression is strongly induced, while ChREBP-α expression is downregulated, totally leading to lipo-glucotoxicity to β-cells, which causes β-cell apoptosis and reduced insulin secretion. In skeletal muscle, MondoA is persistently-activated, resulting in inhibition of glucose uptake, but promotion of lipid synthesis. Interestingly, in the liver, only ChREBP-β expression is dramatically induced, which then directly increases hepatic lipid synthesis and indirectly enhances glucose uptake. Strikingly, in adipose tissue, both ChREBP-α and ChREBP-β expression is downregulated, leading to reduced lipid synthesis and glucose uptake. Overall, the sum of this metabolic remodeling causes insulin resistance (IR). The arrow pointing up means an increase in the level or activity, the arrow pointing down means a decrease in the level or activity, and the bold arrow means the range of increase or decrease are enhanced. The cartoons used in this figure are adapted from the internet.

Skeletal muscle represents about 40% of body mass, but accounts for 80% of glucose uptake in the body, therefore it plays a fundamental role in the regulation of whole-body glucose homeostasis [61]. Accordingly, enhanced skeletal muscle metabolism or exercise can reduce the incidence of metabolic syndrome, hepatic steatosis, and T2D, whereas skeletal muscle dysfunction or physical disability could contribute to hepatic steatosis and T2D [62]. Therefore, a better understanding of the molecular mechanisms that regulate muscle energy homeostasis may reveal new strategies for combating metabolic diseases. Recently, studies have revealed that MondoA is an important negative regulator of muscle insulin sensitivity, which enables MondoA as a potential therapeutic target for IR and T2D [11,63]. The relevant data are summarized in Figure 2 and Table 2.

### 3.1. Role of MondoA in Muscle Glucose Metabolism

As mentioned previously, MondoA is predominantly expressed in skeletal muscle, but its specific roles in skeletal muscle were not clear until recently. By adapting human skeletal myotube as an in vitro model, Ahn et al. have found very recently that MondoA activity in skeletal muscle is physiologically regulated by glucose or fructose level [11]. Specifically, glucose deprivation inhibits, in contrast glucose refeeding activates, the activity of MondoA. In addition, global RNA-seq analysis has further revealed that MondoA activates a wide range of genes, which are involved in lipid metabolism (e.g., acyl-CoA synthetase long chain family member 1 (ACSL1)), elongation of very long chain fatty acids protein 5 (ELOVL5), stearoyl-CoA desaturase (SCD)), glycogen synthesis (e.g., phosphoprotein phosphatase 1 regulatory subunit 3A (PPP1R3A)), and hexosamine biosynthetic pathway (e.g., glutamine-fructose-6-phosphate transaminase (GFPT)-1/2), but strikingly inhibits the expression of genes implicated in insulin signaling pathway (e.g., thioredoxin-interacting protein (TXNIP) and arrestin domain–containing 4 (ARRDC4)) in human skeletal myotubes [11]. Consistent with the RNA-seq results, knockdown of MondoA in human skeletal myotubes inhibits oleate loading-induced triglycerides (TG) accumulation, but enhances 2-deoxyglucose uptake [63]. Notably, the increased glucose uptake is also observed in Mlx-knockdown myoblasts [70], further confirming the inhibitory effects of MondoA on glucose uptake in skeletal muscle. Since the intact insulin signaling is crucial for the insulin-stimulated glucose uptake, and meanwhile, the intracellular glucose level determines MondoA activity, thus it seems that there exists a MondoA-mediated negative feedback mechanism by which MondoA functions as a gatekeeper to maintain glucose homeostasis in skeletal muscle.

### 3.2. Role of Muscle MondoA in IR Development

However, under conditions of chronic nutrient overload, the MondoA-mediated negative feedback mechanism is crashed, leading to persistently activated MondoA, which results in a vicious cycle of muscle lipid accumulation and IR. In this setting, MondoA actually acts as a molecular driver for the pathogenesis of IR. In support, selective loss of MondoA in mice muscle improves high-fat diet (HFD)-induced glucose tolerance and IR through reducing muscle lipid accumulation, and improving muscle insulin signaling as well as glucose uptake [11]. Besides, given the global MondoA-deficient mice show enhanced exercise capacity [64], it is possible that the mice with muscle-specific MondoA deficiency may also improve physical activity, which probably contributes to the improvement of insulin sensitivity as well. Inaddition, mice administrated with a small molecule, SBI-993, which is identified as an inhibitor of MondoA, also exhibit reduced muscle lipid accumulation, as well as improved muscle insulin signaling and systemic glucose tolerance upon HFD feeding [63]. However, it is noteworthy that SBI-993 may also inhibit hepatic ChREBP activity, as the occupation of ChREBP on liver target genes is reduced after SBI-993 treatment [63]. Thus, MondoA-specific inhibitors are needed to further verify the therapeutic potential of MondoA. Nevertheless, the present studies have strongly suggested that the inhibition of MondoA-Mlx transcriptional activity may provide an attractive strategy for the development of anti-diabetic therapeutics.

## 4. Hepatic ChREBP: More Protector than Killer for Insulin Sensitivity

The liver plays a central role in the maintenance of systemic glucose homeostasis in response to different energy status. [71]. Under fasting conditions, liver produces glucose through glycogenolysis and gluconeogenesis to supply the energy for the needs of other organs, e.g., the brain [72]. After feeding, liver stores extra energy in the form of glycogen [72]. In addition, liver is also the principal organ responsible for the conversion of excess dietary carbohydrate into TG through glycolysis and DNL, which is then mobilized and exported to adipose tissue for long-term storage [73]. To maintain the energy balance, these physiological processes in the liver are precisely regulated under normal physiological conditions. However, under conditions of chronic energy excess, the dysregulation of energy metabolism results in the accumulation of TG in the liver, leading to the pathogenesis of nonalcoholic fatty liver disease (NAFLD), IR and T2D [74,75].

ChREBP is most highly expressed in the liver and has emerged as a principal insulin-independent hepatic lipogenic determinant during the last decade [28,55]. A growing number of evidence has shown that ChREBP is involved in the pathogenesis of hepatic steatosis and IR, but the current records are still controversial about the roles of hepatic ChREBP in IR development (reviewed in [76,77]). The relevant data are also summarized in Figure 2 and Table 2.

### 4.1. Promotion of IR Development by Hepatic ChREBP

Hepatic ChREBP expression is highly regulated by nutritional states. Under physiological conditions, the hepatic expression of both ChREBP-α and ChREBP-β is low in fasting state, but it can be significantly induced (especially for ChREBP-β isoform) when refeeding with carbohydrate-rich diet [78]. However, under pathological conditions, the hepatic ChREBP expression is continuously upregulated. For example, compared with the lean controls, the expression of hepatic ChREBP-β (but not ChREBP-α) is dramatically increased in obese humans, which is further elevated in patients with T2D regardless of under fasting or refeeding conditions [78,79,80]. Similar expression patterns are also observed in the livers of HFD-induced obese mice and genetically induced obese *ob/ob* mice [14]. These results indicate a functional relevance of ChREBP in promoting the development of IR and T2D.

In support of this speculation, so far, the most direct evidence is derived from two early studies performed in the *ob/ob* mouse model, where globally or liver-selectively deficiency of ChREBP decreases hepatic lipogenesis and restores hepatic and peripheral insulin sensitivity [14,65]. Consistently, indirect inhibition of ChREBP transcriptional activity by hepatic overexpression of dominant negative Mlx also decreases hepatic steatosis and improves glucose tolerance in diabetes-prone aging C57BL6J mice [81]. Furthermore, the similar phenotypes are also recently observed in mice where hepatic ChREBP expression is suppressed due to deletion of ChREBP upstream regulator retinol saturase (RetSat) or zinc finger and BTB domain-containing protein 20 (ZBTB20) [82,83]. Therefore, it is persuasive that hepatic ChREBP acts as a molecular driver for IR development at least in mice with leptin deficiency.

### 4.2. Inhibition of IR Development by Hepatic ChREBP

Interestingly, recent studies have indicated that ChREBP is beneficial for hepatic insulin sensitivity and whole-body glucose homeostasis. Benhamed et al. have found that mice with liver-specific ChREBP overexpression develop obvious hepatic steatosis, but surprisingly protect against the systemic IR regardless of feeding with normal diet or HFD [67]. Consistently, in patients with nonalcoholic steatohepatitis (NASH), ChREBP expression is positively correlated with the degree of hepatic steatosis, but inversely related to IR [67]. One early explanation for ChREBP-mediated IR improvement is that hepatic upregulation of ChREBP induces the expression of SCD1, which remodels the lipid partitioning in the liver, including raising beneficial lipid species (e.g., monounsaturated fatty acids (MUFAs)) and reducing deleterious lipid species (e.g., saturated fatty acids (SFAs)) [67]. In addition, recent studies have provided an alternative mechanism in which ChREBP directly activates the expression of fibroblast growth factor 21 (FGF21) [34,84,85], an important hepatokine that plays a beneficial role in whole-body metabolic regulation [86,87]. Thus, it seems that the mechanisms for the improvement of IR by ChREBP overexpression is complex. Besides, in agreement with the ChREBP overexpression studies, liver-specific ChREBP knockout mice show dampened hepatic insulin sensitivity, elevated hepatic glucose production, and impaired systemic glucose tolerance under various feeding conditions, including normal chow and HFD [66]. Moreover, the global ChREBP deficient mice also exhibit IR and glucose intolerance, albeit multiple tissues involvement [55]. Thereby, hepatic ChREBP indeed protects against IR development at least under the above-mentioned conditions.

Overall, the role of hepatic ChREBP in IR development seems to be context-dependent, but under most conditions, ChREBP is more likely protective rather than detrimental in maintaining insulin sensitivity. However, given the fact that overexpression of ChREBP also causes hepatic steatosis, which impairs the insulin sensitivity, thus activation of ChREBP may be not a good choice for the improvement of hepatic insulin sensitivity.

## 5. Adipose ChREBP: A Master Regulator of Systemic Insulin Sensitivity

Adipose tissues (ATs) play an important role in regulating whole-body energy and glucose homeostasis [88]. There are two principal types of ATs, namely white adipose tissue (WAT) and brown adipose tissue (BAT). The WAT stores extra energy from the diets in the form of TG, which can be mobilized as free acids to meet the energy demand of other tissues in states of fasting or exercise. In addition, WAT also secretes diverse adipokines and lipokines in response to different metabolic stress, and functions as an important endocrine organ [89]. In contrast, BAT is specialized for energy expenditure, which burns lipids for heat production as a defense against cold and obesity [90]. With the global increase in the prevalence of obesity, ATs have attracted much attention concerning its role in the development of IR and T2D. In various causes of IR, the impaired DNL in ATs has emerged as an important player in recent years [91,92]. ChREBP, as a major determinant for adipose DNL, is therefore considered to be a promising target to reverse IR [60,93]. The relevant data are summarized in Figure 2 and Table 2.

### 5.1. The Disturbed Expression of Adipose ChREBP during IR Development

Opposite with that in the liver, ChREBP expression in ATs is often impaired under various detrimental conditions. Specifically, the short-term HFD, which causes IR, reduces DNL and ChREBP expression in both WAT and BAT [56,94]. That occurs even earlier than the impairment of phosphorylation of AKT (also known as protein kinase B, PKB), a key step during the pathogenesis of IR [94]. The similar results are also observed in WATs of the *ob/ob* mice as young as seven-weeks old [94]. Therefore, it is more likely that the repressed expression of adipose ChREBP might be the cause but not the consequence of the IR development. Consistent with animal studies, in adolescents with prediabetes or early T2D, the subcutaneous adipose tissue (SAT) expresses lower amounts of ChREBP-α/β compared with that in the healthy controls [95]. Besides, in adult obese subjects either with or without T2D, ChREBP-α is also lower-expressed in the SAT, but for the ChREBP-β level, it is still uncertain due to its very low expression [80]. Interestingly, in the visceral adipose tissue (VAT), only the ChREBP-β expression is downregulated in obese individuals, implying existence of a tissue-specific regulation of ChREBP-α/β expression [80]. Moreover, ChREBP-β expression in VAT is positively correlated with the VAT DNL, but reversely related to homeostasis model assessment of insulin resistance (HOMA-IR) and liver steatosis in adult subjects [80]. Thereby, the expression levels of ChREBP in ATs could be used as an early predictor for IR development.

### 5.2. Maintenance of Systemic Insulin Sensitivity by WAT ChREBP

Consistent with the ChREBP expression during the pathogenesis of IR, the gain/loss-of-function studies in animals have further demonstrated that WAT ChREBP preserves the systemic insulin sensitivity. Specifically, mice with fat-specific loss of ChREBP show whole-body IR and WAT inflammation regardless of the lean or obese state [12]. Consistently, global ChREBP knockout mice also exhibit impaired insulin sensitivity [55]. At the molecular level, ChREBP deficiency in adipocytes alters fatty acid composition and lowers the abundance of the newly-identified palmitic acid esters of hydroxy stearic acids (PAHSAs) in WATs [12]. PAHSAs are synthesized through the process of WAT DNL and function as an insulin-sensitizing fatty acid through multiple mechanisms, including stimulating glucose uptake and lowering inflammation in ATs, and also including inducing the secretion of intestinal glucagon-like peptide-1 (GLP-1) and pancreatic insulin [96]. Because of that, supplementation of 9-PAHSA, the most abundant PAHSA isomer in WATs and serum, restores insulin sensitivity and reverses WAT inflammation in fat-specific ChREBP deficient mice [12]. In line with the loss-of-function studies, transgenic overexpression of constitutively active ChREBP (ChREBP-CA) in mice ATs improves insulin sensitivity and glucose tolerance under western diet conditions [68]. Since the levels of PAHSAs are not measured in these transgenic mice, it is still uncertain about the contributions of PAHSAs in the improvement of insulin sensitivity of these mice. In addition to regulating the DNL pathway, it is noteworthy that ChREBP also has other functions in WATs, such as promoting adipocytes differentiation [97] and white adipocyte browning [98], which are both known to positively regulate systemic insulin sensitivity [99,100]. Therefore, WAT ChREBP improves systemic insulin sensitivity probably through multiple mechanisms.

Consistent with the beneficial effects of ChREBP on systemic insulin sensitivity, genetic manipulations of ChREBP upstream factors or binding partners in ATs also regulate mice insulin sensitivity. Glucose transporter 4 (GLUT4) is responsible for the glucose entering into the adipocytes, while glucose metabolites can activate ChREBP, therefore, alterations in adipose GLUT4 expression have profound effects on systemic insulin sensitivity through regulation of ChREBP-α-mediated expression of ChREBP-β [60]. Besides, HSL has been recently identified to be a direct binding partner of ChREBP in adipocytes [52]. The physical interaction between ChREBP-α and HSL keeps ChREBP-α to stay in the cytosol, which blocks ChREBP-α translocation into the nucleus to induce the expression of ChREBP-β [52]. Thus, genetic loss of HSL in mouse ATs can release ChREBP-α, leading to enhanced mice insulin sensitivity, albeit the mice concurrently develop hepatic steatosis [52,101]. Overall, these studies strongly suggest that the WAT ChREBP is a master regulator of systemic insulin sensitivity.

### 5.3. BAT ChREBP is Dispensable for Systemic Insulin Sensitivity

Although there are no documents directly depicting BAT ChREBP function in the regulation of systemic insulin sensitivity to date, it is already known that BAT DNL seemingly has limited effects on whole-body insulin sensitivity. Abolition of BAT DNL by the specific loss of fatty acid synthase (FASN, the key rate-limiting enzyme in DNL) in brown adipocytes do not alter mice glucose tolerance regardless of feeding with normal chow or HFD [94]. In particularly, loss of AKT2 selectively in BAT impairs the expression and activity of ChREBP, leading to BAT lipodystrophy as expected, but leaves insulin sensitivity and glucose tolerance intact [56]. Together, these results suggest that BAT ChREBP seems to be not essential for the regulation of systemic insulin sensitivity, but more direct evidence is still needed to this end.

Collectively, ChREBP in ATs, particularly in WATs, plays an important role in the maintenance of systemic insulin sensitivity. Since the expression of ChREBP, especially the ChREBP-β isoform, is often downregulated in conditions predisposing to diabetes, therefore, restoration of ChREBP expression or activity in WATs might be an important therapeutic strategy to treat T2D and its complications.

## 6. Pancreatic ChREBP: A Double-Edged Sword for Insulin Production

Pancreatic β-cells play an important role in maintaining levels of circulating glucose constant through the production of insulin in response to nutritional states [102]. The production of insulin is tightly regulated by physiological cues. Usually, feeding activates, while fasting blocks, insulin production via regulation of β-cell proliferation and insulin gene transcription [102]. However, under conditions of chronic overnutrition, the resulting hyperglycemia causes progressive and deleterious effects on β-cells, leading to reduced insulin production and subsequent β-cells failure, a hallmark of T2D [103]. Thus, understanding the molecular mechanisms responsible for the regulation of β-cells growth and failure is necessary for developing new drugs to cure T2D. In recent years, ChREBP has been revealed to be implicated in both β-cell proliferation and apoptosis [13,69,104], enabling it as a potential target for treating T2D. The relevant data are also summarized in Figure 2 and Table 2.

### 6.1. Role of Pancreatic ChREBP in β-cell Adaptive Proliferation

Both ChREBP-α and ChREBP-β are expressed in pancreatic β-cells, and their levels/activities are induced by high glucose concentration [13,104]. Additionally, using gain/loss-of-function studies, ChREBP-α/β has been revealed to be both necessary and sufficient for glucose-induced β-cell adaptive proliferation in vitro [13,104]. Specifically, adenoviral overexpression of wild-type ChREBP-α in rat or human β-cells augments glucose-stimulated β-cell proliferation, and meanwhile increases the expression of cell cycle accelerators, including cyclin D2, cyclin A, cyclin E, and cyclin-dependent kinase (CDK) 4/6 [13]. Reversely, depletion of total ChREBP or ChREBP-β alone by small interfering RNA (siRNA) blocks glucose-induced β-cell proliferation and expression of cyclin A and cyclin E [13,104]. The proposed mechanisms include at least two waves of transcriptional cascades [105]. Glucose initiates the first wave of transcription during which activated-ChREBP-α is recruited to a tissue-specific element in the ChREBP-β promoter to activate the expression of ChREBP-β, which then further enhances β-cell glucose and lipid metabolism [104,105]. Thereafter, the expression of cell-cycle genes are induced in the second wave, where ChREBP activates several cell cycle drivers, including nuclear receptor retinoic acid receptor-related orphan receptor-γ (ROR-γ), to promote β-cell proliferation [105]. Besides, more recently, nuclear factor erythroid 2–related factor 2 (NRF2) has been also revealed to be implicated in ChREBP-mediated β-cell proliferation [106]. ChREBP-α activation of NRF2 in β-cells enhances the antioxidant and mitochondrial biogenic programs, which accelerate β-cell proliferation through supplying sufficient energy [106]. Overall, these findings suggest that ChREBP is a central regulator in glucose-induced β-cell adaptive proliferation and highlight ChREBP as a potential therapeutic target for β-cell regeneration in patients with T2D. However, to date the relevant in vivo studies are still missing and should be warranted in the future.

### 6.2. Role of Pancreatic ChREBP in β-Cell Failure

Strikingly, ChREBP has also been revealed to be a key contributor to pancreatic β-cell dysfunction under conditions of chronic caloric excess. Firstly, the pancreatic ChREBP is activated in conditions susceptible to T2D. Specifically, the total protein levels of ChREBP, particularly the nucleus-localized ChREBP, are significantly increased in human diabetic pancreatic islets compared with non-diabetic controls [69,107]. Similarly, in the mice model of diabetes, including NOD mice and *ob/ob* mice (which represents type 1 and type 2 diabetes mouse model, respectively), the ChREBP-β mRNA level is dramatically induced compared with the lean mice or non-diabetic controls [108]. But interestingly, the ChREBP-α mRNA level is downregulated under the same conditions, which may act as a negative feedback mechanism to limit the excessive glucose-induced gene expression in β-cells [108].

Secondly, the activation of pancreatic ChREBP is more likely the cause for the development of T2D. As happened in prolonged hyperglycemia in diabetes, overexpression of ChREBP-CA via viral vectors for too much or too long, causes β-cell apoptosis in vitro [69]. Consistently, β-cell-specific overexpression of ChREBP-CA in mice is sufficient to induce diabetic phenotypes, including β-cell apoptosis and hyperglycemia [69]. At the molecular level, the persistently activated-ChREBP increases β-cell lipid synthesis, and meanwhile decreases mitochondrial fatty acid β-oxidation (through inducing the expression of glycolic and lipogenic genes [69], but inhibiting the expression of peroxisome proliferator-activated receptor α (PPARα) [109], respectively), resultantly leading to increase of intracellular lipid accumulation and ultimately causing lipotoxicity to β-cells. In parallel, the activated ChREBP also induces the expression of TXNIP [110], a critical factor involved in glucose-induced inflammation, oxidative stress as well as apoptosis in β-cells [111,112], thus causing glucotoxicity to β-cells. Therefore, the lipotoxicity and glucotoxicity (also termed as lipo-glucotoxicity) together ultimately lead to β-cell failure [69]. In support, inhibition or deletion of TXNIP in mouse β-cells protects against β-cell apoptosis and diabetes [111]. Meanwhile, the increase of TXNIP expression by selective loss of mechanistic target of rapamycin (mTOR) in mouse β-cells exacerbates the development of diabetes [107]. However, the above speculations are not verified in β-cell-specific ChREBP deficient mice, which should be tested in the future.

Collectively, pancreatic ChREBP plays dual roles in the pathogenesis of T2D. In response to the acute high glucose stimulation, pancreatic ChREBP protects against hyperglycemia through promoting β-cell proliferation. However, under conditions of chronic nutrient overload, the continuously activated ChREBP results in β-cell apoptosis, which exacerbates T2D development. Thereby, developing strategies to precise regulation of ChREBP expression and/or activity in pancreatic β-cells may represent a therapeutic approach in the treatment of T2D.

## 7. Conclusions and Perspectives

In summary, recent studies have revealed that glucose-sensing transcription factor MondoA/ChREBP plays a fundamental role in the control of systemic glucose metabolism and insulin sensitivity via a context- and tissue-dependent manner. Under normal physiological conditions, MondoA/ChREBP acts as a key player in each metabolic tissue to synergistically maintain the whole-body glucose homeostasis and energy metabolism. However, under conditions of chronic energy surplus, the dysregulated expression and/or activity of MondoA/ChREBP in metabolic tissues often results in a common consequence, namely IR, a prerequisite for the development of T2D. Therefore, targeting MondoA/ChREBP might offer new approaches to combat T2D and its complications in humans.

Based on the functional characteristic of MondoA/ChREBP, so far, at least two strategies can be adopted to develop drugs to prevent T2D. One aim is to develop MondoA/ChREBP-specific inhibitors. This strategy is supported by one recent study in which SBI-993, an inhibitor of MondoA and probably also ChREBP, was given to mice and alleviated HFD-induced mice IR [63]. However, the challenge of this strategy is that the crystal structures of the two proteins (or at least of the glucose sensing module (GSM)) are still unknown. Thus, elucidating the structural basis of the GSM will be required in the future. In addition, given the fact that the inhibition of MondoA/ChREBP in the muscle and pancreas has opposing effects with that in the WAT and the liver, thereby the effectiveness of this strategy might be weakened due to the functional disparities among the different metabolic tissues. Moreover, MondoA and ChREBP are widely expressed, and their functions have not been clearly examined in other tissues, e.g., the brain, kidney, and intestine. Therefore, the systemic evaluation of the consequences of MondoA/ChREBP inhibition will be necessary before the inhibitors are applied to clinical practices.

To avoid the shortcomings of the first strategy, the second strategy focuses on the tissue-specific MondoA/ChREBP targets or effectors which have potentials to serve as the drug leads. One such example is that the insulin-sensitizing fatty acids PAHSAs, which are synthesized by ChREBP-mediated DNL in ATs, have been recently shown with great therapeutic potentials to treating T2D [96]. Thus, this strategy seems to be more promising than the first one, but currently it is still hard to follow due to the lack of the detailed molecular atlas in each metabolic tissue. Hence, more mechanistic studies are still required in the future, especially including the ones in which new technologies, such as mass spectrometry and lipidomics [113], are adopted to deepen our understanding of the downstream events of MondoA/ChREBP.

## Figures and Tables

**Table 1:** Summary of characteristic differences between MondoA and ChREBP.

Characteristic	MondoA	ChREBP
Other names	MLXIP	MLXIPL, MondoB, WBSCR14
Coding gene location (*Homo sapiens*)	chromosome 12q24.31	chromosome 7q11.23
Isoforms	MondoA	ChREBP-α and ChREBP-β
Protein weight (*Homo sapiens*)	919 AA	852 AA and 675AA
Primary enriched tissues	skeletal muscle	liver, adipose tissue
Basal subcellular localization	outer mitochondrial membrane	cytosol
Major downstream pathways	glycolysis	lipogenesis

**Table 2:** Summary of the reported tissue- and context-dependent roles of MondoA/ChREBP in mice insulin sensitivity.

Mouse Models	Context	Body Weight	Fat Mass	Hepatic Steatosis	Insulin Sensitivity	Reference
MondoA global knockout	Standard diet	=	ND	ND	=	[64]
	High-fat diet	NA				NA
MondoA muscle-specific knockout	Standard diet	=	ND	ND	=	[11]
	High-fat diet	=	ND	ND	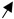	[11]
ChREBP global knockout	Standard diet	=	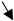	=	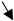	[55]
	Standard diet in *ob/ob* mice background	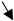	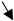	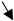	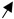	[65]
ChREBP liver-specific knockout	Standard diet	=	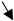	=	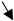	[66]
	High-fat diet	=	=	=	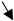	[66]
	High-carbohydrate diet	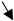	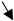	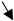	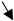	[66]
ChREBP liver-specific overexpression	Standard diet	=	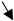	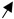	=	[67]
	High-fat diet	=	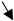	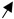	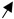	[67]
ChREBP AT-specific knockout	Standard diet	=	=	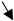	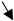	[12]
	High-fat diet	=	=	=	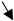	[12]
ChREBP AT-specific overexpression	Standard diet	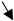	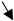	=	=	[68]
	High-fat diet	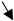	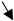	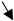	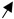	[68]
ChREBP pancreatic β cell-specific overexpression	Standard diet	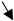	ND	ND	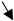	[69]
	High-fat diet	NA				NA

Note: “=” means not changed. ND = not detected. NA = not available. The arrow pointing up means an increase in the level. The arrow pointing down means a decrease in the level.

## Abbreviations

ACSL1	acyl-CoA synthetase long chain family member 1
AMPK	AMP-activated protein kinase
ARRDC4	arrestin domain–containing 4
AT	adipose tissue
BAT	brown adipose tissue
CBP	CREB-binding protein
ChoRE	carbohydrate response element
ChREBP	carbohydrate response element-binding protein
ChREBP-CA	constitutively active form of ChREBP
CRM1	chromosomal region maintenance 1
CDK4/6	cyclin-dependent kinase (CDK) 4/6
DNL	de novo lipogenesis
ELOVL5	elongation of very long chain fatty acids protein 5
FASN	fatty acid synthase
FGF21	fibroblast growth factor 21
Fru-2,6-P2	fructose-2,6-bisphosphate
FXR	farnesoid X receptor
G6P	glucose-6-phosphate
GCGR	glucagon receptor
GFPT1/2	glutamine-fructose-6-phosphate transaminase 1/2
GLP-1	glucagon-like peptide-1
GLUT	glucose transporter
GSM	glucose sensing module
HCF-1	host cell factor-1
HFD	high-fat diet
HNF-4α	hepatocyte nuclear factor-4α
HOMA-IR	homeostasis model assessment of insulin resistance
HSL	hormone-sensitive lipase
IR	insulin resistance
MCP-1	monocyte chemoattractant protein-1
Mlx	Max-like protein x
MLXIP	Mlx-interacting protein
MLXIPL	Mlx-interacting protein-like
mTOR	mechanistic target of rapamycin
MUFAs	monounsaturated fatty acids
NAFLD	nonalcoholic fatty liver disease
NASH	nonalcoholic steatohepatitis
NRF2	nuclear factor erythroid 2-related factor 2
OGT	O-linked N-acetylglucosamine transferase
PAHSAs	palmitic acid esters of hydroxy stearic acids
PGC-1β	peroxisome proliferator-activated receptor-γ coactivator-1β
PHF2	PHD finger protein 2
PKA	protein kinase A
PPARα	peroxisome proliferator-activated receptor-α
PPARγ	peroxisome proliferator-activated receptor-γ
PPP1R3A	phosphoprotein phosphatase 1 regulatory subunit 3A
RetSat	retinol saturase
RORγ	retinoic acid receptor-related orphan receptor-γ
SAT	subcutaneous adipose tissue
SCD	stearoyl-CoA desaturase
SFAs	saturated fatty acids
TG	triglycerides
TNFα	tumor necrosis factor-α
TXNIP	thioredoxin-interacting protein
VAT	visceral adipose tissue
WAT	white adipose tissue
Xu5P	xylulose 5-phosphate
T2D	type 2 diabetes
ZBTB20	zinc finger and BTB domain-containing protein 20
